# Avelumab treatment in Italian patients with metastatic Merkel cell carcinoma: experience from an expanded access program

**DOI:** 10.1186/s12967-021-02730-8

**Published:** 2021-02-15

**Authors:** Giovanni Grignani, Vanna Chiarion Sileni, Carmine Pinto, Roberta Depenni, Nicola Fazio, Luca Galli, Dario Giuffrida, Carlo Carnaghi, Domenico Ciliberto, Domenico C. Corsi, Paola Queirolo, Elena Benincasa, Filippo Venturini, Gennaro Fazzi, Nuno Costa, Paolo Antonio Ascierto

**Affiliations:** 1 Candiolo Cancer Institute, FPO - IRCCS, Candiolo (TO) 10060, Italy; 2 Istituto Oncologico Veneto, Istituto di Ricovero e Cura a Carattere Scientifico, Padua, Italy; 3 Medical Oncology Unit, Clinical Cancer Centre, IRCCS–AUSL di Reggio Emilia, Reggio Emilia, Italy; 4 University Hospital of Modena and Reggio Emilia, Modena, Italy; 5 European Institute of Oncology, IEO, IRCCS, Milan, Italy; 6 Department of Medical Oncology, Azienda Ospedaliero-Universitaria Pisana and University of Pisa, Istituto Toscano Tumori, Pisa, Italy; 7 Department of Medical Oncology, Istituto Oncologico del Mediterraneo, Viagrande, Italy; 8 Division of Medical Oncology, Ospedale Centrale di Bolzano, Bolzano, Italy; 9 Medical Oncology Unit, Department of Experimental and Clinical Medicine, Magna Graecia University, Catanzaro, Italy; 10 Medical Oncology Unit, Ospedale San Giovanni Calibita, Fatebenefratelli, Rome, Italy; 11 Division of Medical Oncology for Melanoma, Sarcoma, and Rare Tumors, European Institute of Oncology, IEO, IRCCS, Milan, Italy; 12 Merck KGaA, Darmstadt, Germany; 13 Merck Serono SpA, Rome, Italy; an affiliate of Merck KGaA, Darmstadt, Germany; 14 Pfizer Inc, Porto Salvo, Portugal; 15 Istituto Nazionale Tumori IRCCS Fondazione G. Pascale, Naples, Italy

**Keywords:** Avelumab, Merkel cell carcinoma, Second line, Expanded access program, PD-L1

## Abstract

**Background:**

The incidence of Merkel cell carcinoma (MCC), a rare form of skin cancer with a poor prognosis, has increased in Italy in recent decades. Avelumab, an anti-programmed death ligand 1 monoclonal antibody, is approved for the treatment of metastatic MCC (mMCC) based on the results of the phase 2 JAVELIN Merkel 200 trial. The global avelumab expanded access program (EAP) was designed to provide compassionate use of avelumab prior to approval for patients with mMCC who had limited treatment options. We report findings from a subgroup of Italian patients enrolled in the avelumab EAP.

**Methods:**

Eligible patients had mMCC and progressive disease following ≥ 1 prior line of chemotherapy or were ineligible for chemotherapy or clinical trial participation. Patients received avelumab 10 mg/kg intravenously every 2 weeks. Treating physicians were provided with an initial 3-month supply of avelumab; resupply was permitted if the patient achieved a complete response, partial response, stable disease, or other clinical benefit per physician assessment. Safety and efficacy data for the EAP were reported at the treating physician’s discretion.

**Results:**

Between April 1, 2016, and September 14, 2018, 109 requests for avelumab were received from Italy, and 102 were approved. All but 1 of the approved patients had received ≥ 1 prior line of therapy. At data cutoff (March 22, 2019), 95 patients had been supplied with avelumab and response data were available for 55 patients. The objective response rate in response-evaluable patients was 29.1%, including 6 patients (10.9%) who achieved a complete response and 10 patients (18.2%) who achieved a partial response; in the total population supplied with avelumab (n = 95), the proportion who had an objective response was 16.8%. The median duration of treatment in responding patients was 9.7 months (range, 3.5–41.7 months). The most frequently reported treatment-related adverse events were infusion-related reaction (single preferred term; n = 3 [3.2%]) and pyrexia (n = 2 [2.1%]).

**Conclusions:**

Results from Italian patients enrolled in the avelumab EAP are consistent with the findings of the JAVELIN Merkel 200 trial and confirm the efficacy and safety of avelumab treatment in this population.

## Introduction

Merkel cell carcinoma (MCC) is a rare and aggressive skin cancer [1]. Compared with other skin cancers, particularly melanoma, MCC is rarer and is associated with a worse prognosis; the 10-year overall survival rate for MCC is 18% vs. 61% for melanoma [2]. Risk factors for MCC include UV radiation exposure, advanced age, and a weakened immune system [1]. Approximately 80% of MCC cases are associated with clonal integration of the Merkel cell polyomavirus (MCPyV) [3].

The incidence of MCC has increased in Europe in recent decades, with Italy among the countries with the highest increase in MCC among men [4]. MCC can grow and metastasize quickly, and 6% to 8% of patients have distant metastatic disease at diagnosis [5–7]. Patients with metastatic MCC (mMCC) have limited treatment options and a poor prognosis; median survival duration with chemotherapy is approximately 10 months [1, 8]. European guidelines, last published in 2015, state that apart from surgical removal of isolated metastases, there is no established curative treatment for mMCC [9].

Immune checkpoint inhibitors that block the interaction between programmed cell death protein 1 and its ligand, PD-L1, have received regulatory approval for the treatment of patients with mMCC [1]. In particular, avelumab became the first approved treatment for patients with mMCC in 2017 based on results from JAVELIN Merkel 200, a phase 2, single-arm trial (NCT02155647) [10]. After 3 years of follow-up from part A of the trial, which enrolled 88 patients with progressive disease (PD) who had received prior chemotherapy, the objective response rate (ORR) was 33.0% (95% CI 23.3–43.8%), including complete response (CR) in 11.4% [11]. After ≥ 15 months of follow-up in part B, in which 116 patients with mMCC and no prior systemic treatment for metastatic disease were treated with avelumab, 30.2% of patients had a response lasting ≥ 6 months (durable response rate), and the ORR was 39.7% (95% CI 30.7–49.2%), including CR in 16.4% [12]. In both parts A and B of the trial, responses were seen irrespective of PD-L1 or MCPyV status; however, numerically higher ORRs were reported in patients with PD-L1+ vs. PD-L1− tumors (part A, PD-L1+ [n = 57]: 36.8% [95% CI 24.4–50.7%] and PD-L1− [n = 16]: 18.8% [95% CI 4.0–45.6%]; part B, PD-L1+ [n = 21]: 61.9% [95% CI 38.4–81.9%] and PD-L1− [n = 87]: 33.3% [95% CI 23.6–44.3%]) [11, 12]. Avelumab has subsequently been approved in various countries worldwide for the treatment of mMCC, including in Europe [13].

For patients with no comparable or satisfactory treatment options, expanded access programs (EAPs), also called “compassionate use programs,” allow access to investigational drugs, biologics, and medical devices outside of a clinical trial [14]. The avelumab EAP was an ad hoc program designed to provide compassionate use of avelumab to patients with mMCC with limited treatment options, and participation was permitted on a patient-by-patient basis. Overall results from the global population have been reported previously [15]. This manuscript reports data from a large subgroup of patients enrolled in Italy.

## Methods

To be eligible for participation in the avelumab EAP, patients were required to be ineligible for participation in any ongoing clinical trial for advanced MCC, have an Eastern Cooperative Oncology Group performance status of 0 to 3, and have measurable disease according to Response Evaluation Criteria in Solid Tumors version 1.1 (RECIST 1.1). Patients were also required to have either PD following ≥ 1 prior line of chemotherapy or to be ineligible to receive chemotherapy in the metastatic setting. Patient selection was not based on tumor PD-L1 expression or MCPyV status. Eligibility criteria permitted entry to the EAP for patients with treated brain metastases (without steroid use) that were not progressing or patients who were potentially immunocompromised, evaluated on a case-by-case basis; data for all immunocompromised patients enrolled in the EAP were summarized in a previous report [15].

Treating physicians were provided with a 3-month supply of avelumab, which was administered to patients at a dose of 10 mg/kg by 1-h intravenous infusion every 2 weeks. Patients received avelumab until confirmed PD, unacceptable toxicity, or other criteria for withdrawal occurred; continuation of avelumab beyond radiological PD was permitted on a case-by-case basis in the absence of significant clinical deterioration and based on physician assessment of potential clinical benefit. Patients also received premedication with antihistamine and acetaminophen to mitigate infusion-related reactions, consistent with the summary of product characteristics for avelumab [13]. Resupply of avelumab was allowed for patients with a CR, partial response, or stable disease according to RECIST 1.1 or other clinical benefit, assessed by the treating physician. Physician assessments included best overall response according to RECIST 1.1, duration of treatment for patients with response, safety, and tolerability, and data were collected prospectively. Data were provided at the treating physician’s discretion, and confirmation that supplied avelumab was administered to patients was not required.

All adverse events (AEs), including nonserious and serious AEs, were reported by treating physicians to a global safety database (Merck KGaA, Darmstadt, Germany Global Patient Safety), to the local health unit, and to the ethics committee at the time of resupply, progression, or death. Infusion-related reactions were defined according to a prespecified list of Medical Dictionary for Regulatory Activities terms and managed per established guidance for avelumab [13]. Immune-related AEs were identified by medical review. An online portal to process EAP requests and collate responses was implemented in May 2017.

## Results

Between April 1, 2016, and September 14, 2018, 109 requests for avelumab were received from Italy. A total of 102 were approved, 2 were withdrawn before approval; additionally, 7 requests were withdrawn after approval but before supply and 5 patients did not initiate avelumab treatment. Among approved patients, the median age was 70.6 years (range, 41.0–92.0 years), and all but 1 approved patient (n = 101) were approved to receive avelumab as second-line or later treatment (i.e., had received prior chemotherapy; 1 patient was approved to receive first-line avelumab treatment; Table 1). The data cutoff was March 22, 2019.

**Table 1:** Baseline characteristics of approved patients enrolled in Italy in the avelumab MCC EAP

Characteristic	n = 102
Age	
Median (range), years	70.6 (41.0–92.0)
< 65, n (%)	27 (26.5)
≥ 65, n (%)	75 (73.5)
Sex, n (%)	
Female	23 (22.5)
Male	78 (76.5)
Data missing	1 (1.0)
ECOG PS, n (%)	
0	52 (51.0)
1	30 (29.4)
2	4 (3.9)
3	1 (1.0)
Data missing	15 (14.7)
Line of therapy, n (%)	
1	1 (1.0)
≥ 2	101 (99.0)

*EAP* expanded access program, *ECOG PS* Eastern Cooperative Oncology Group performance status, *MCC* Merkel cell carcinoma

**Fig. 1:**
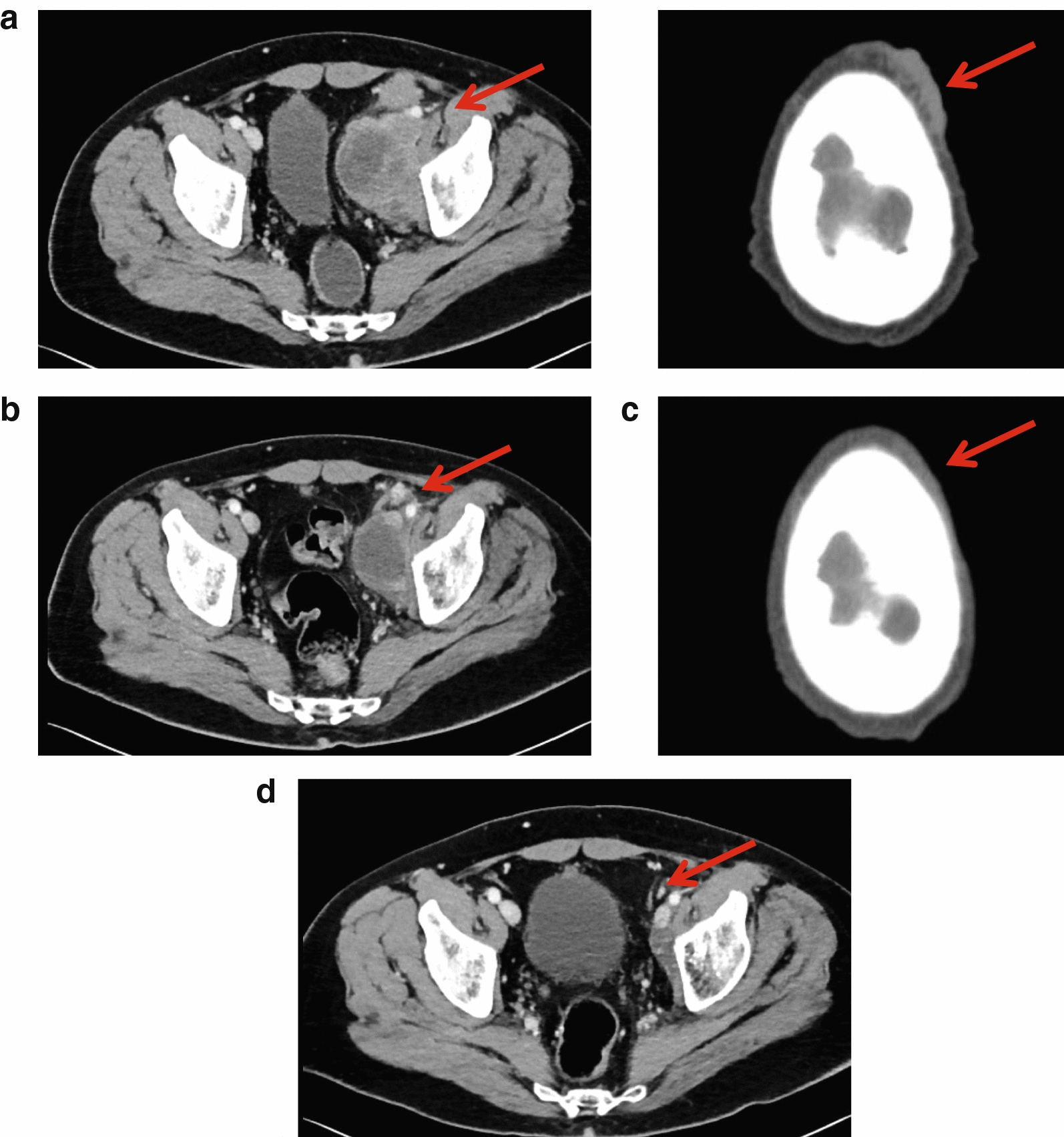
Computed tomography scans from a patient with metastatic Merkel cell carcinoma treated with avelumab. Stable disease at **a** baseline and **b** 2 months, and complete response at **c** 4 months and **d** 1 year after starting avelumab treatment. Images were provided by Dr. Grignani

**Fig. 2:**
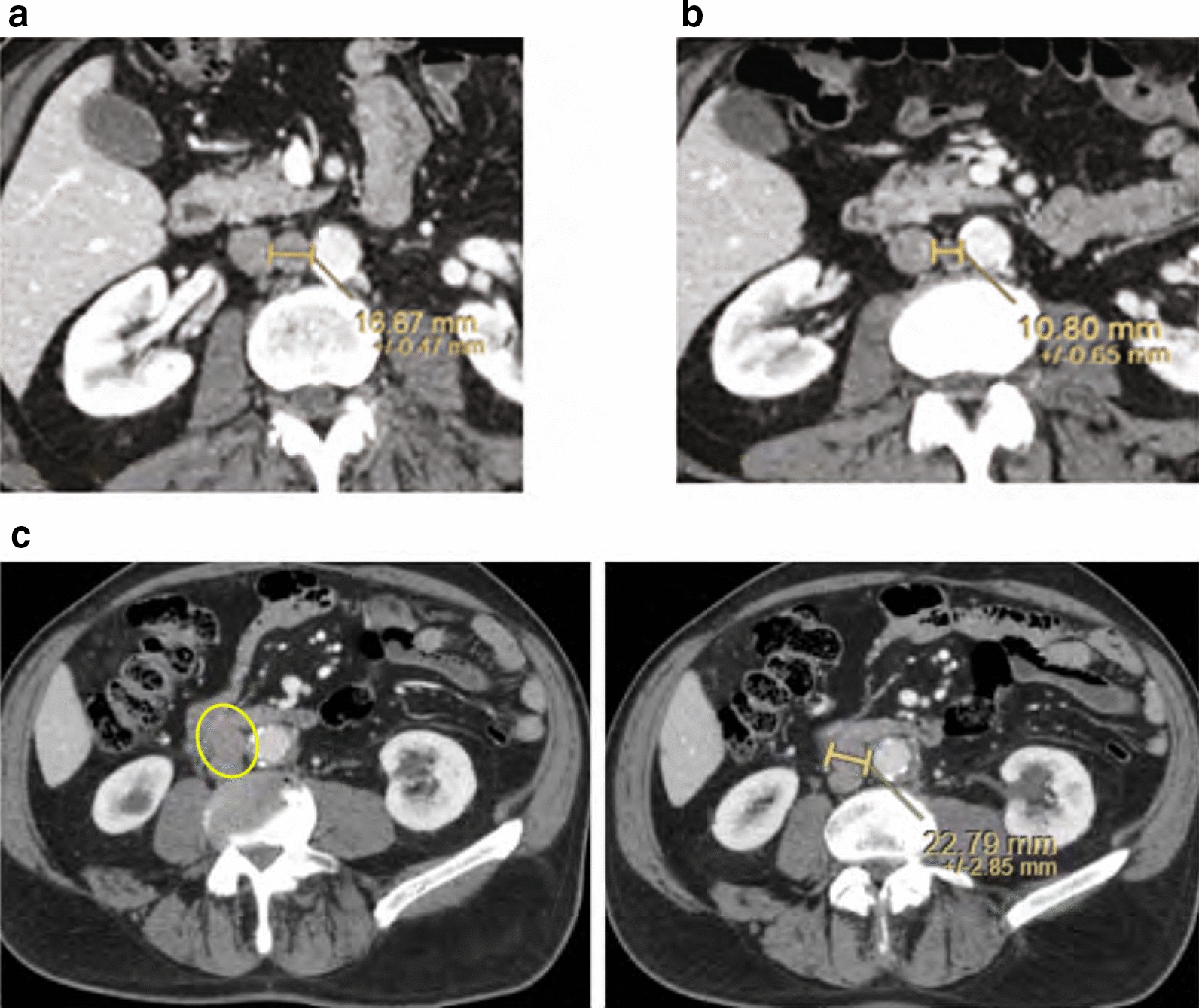
Computed tomography scans from a patient with metastatic Merkel cell carcinoma treated with avelumab. **a** PD at baseline (March 2017), **b** partial response at 18 months after starting avelumab treatment (September 2018), and **c** PD in September 2020 after stopping avelumab treatment in February 2020; the patient has since restarted avelumab treatment. Images were provided by Dr. Chiarion Sileni. *PD* progressive disease

**Fig. 3:**
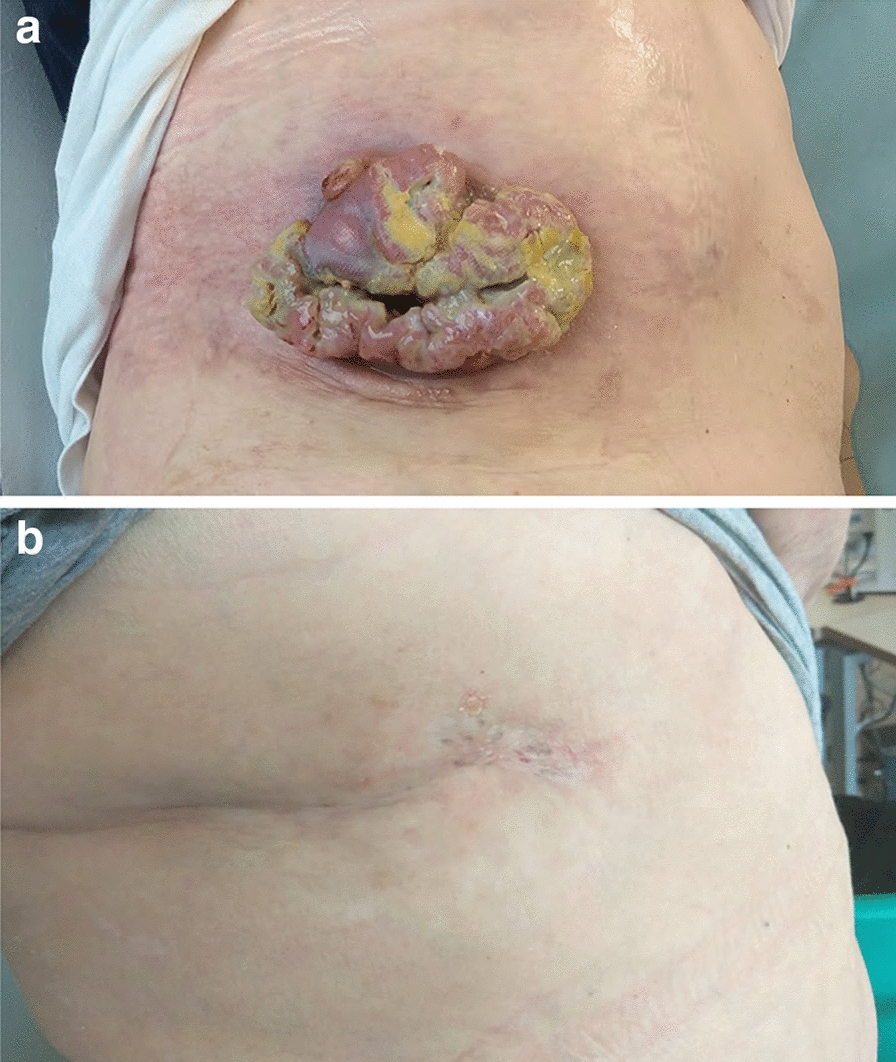
Images from a patient with metastatic Merkel cell carcinoma treated with avelumab, **a** at baseline and **b** 1 year after starting avelumab treatment. Images were provided by Dr. Pinto

**Fig. 4:**
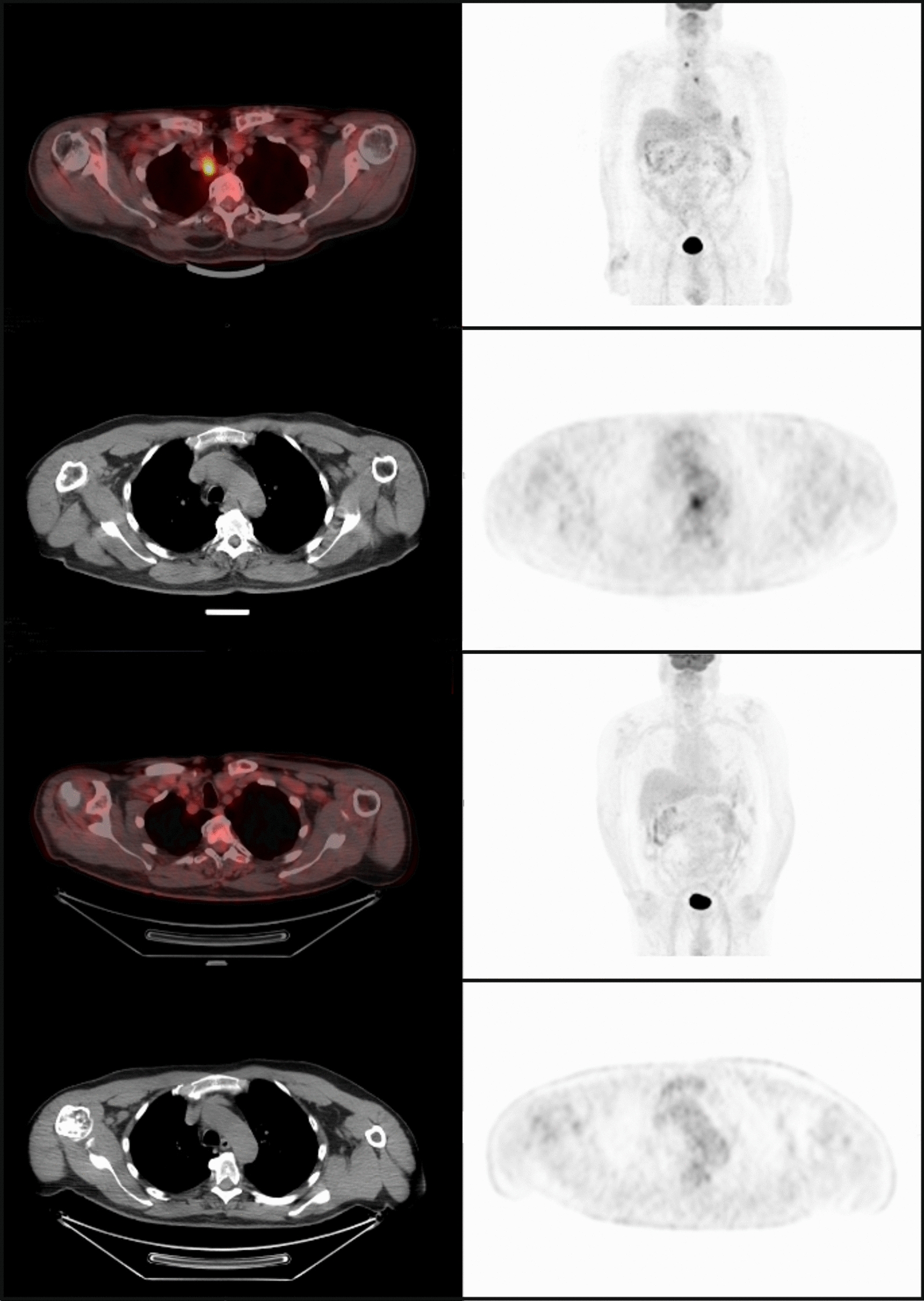
PET-CT scans of a patient with mMCC who achieved a complete response with avelumab. Images were provided by Dr. Carnaghi. *mMCC* metastatic Merkel cell carcinoma, *PET-CT* positron emission tomography–computed tomography

**Fig. 5:**
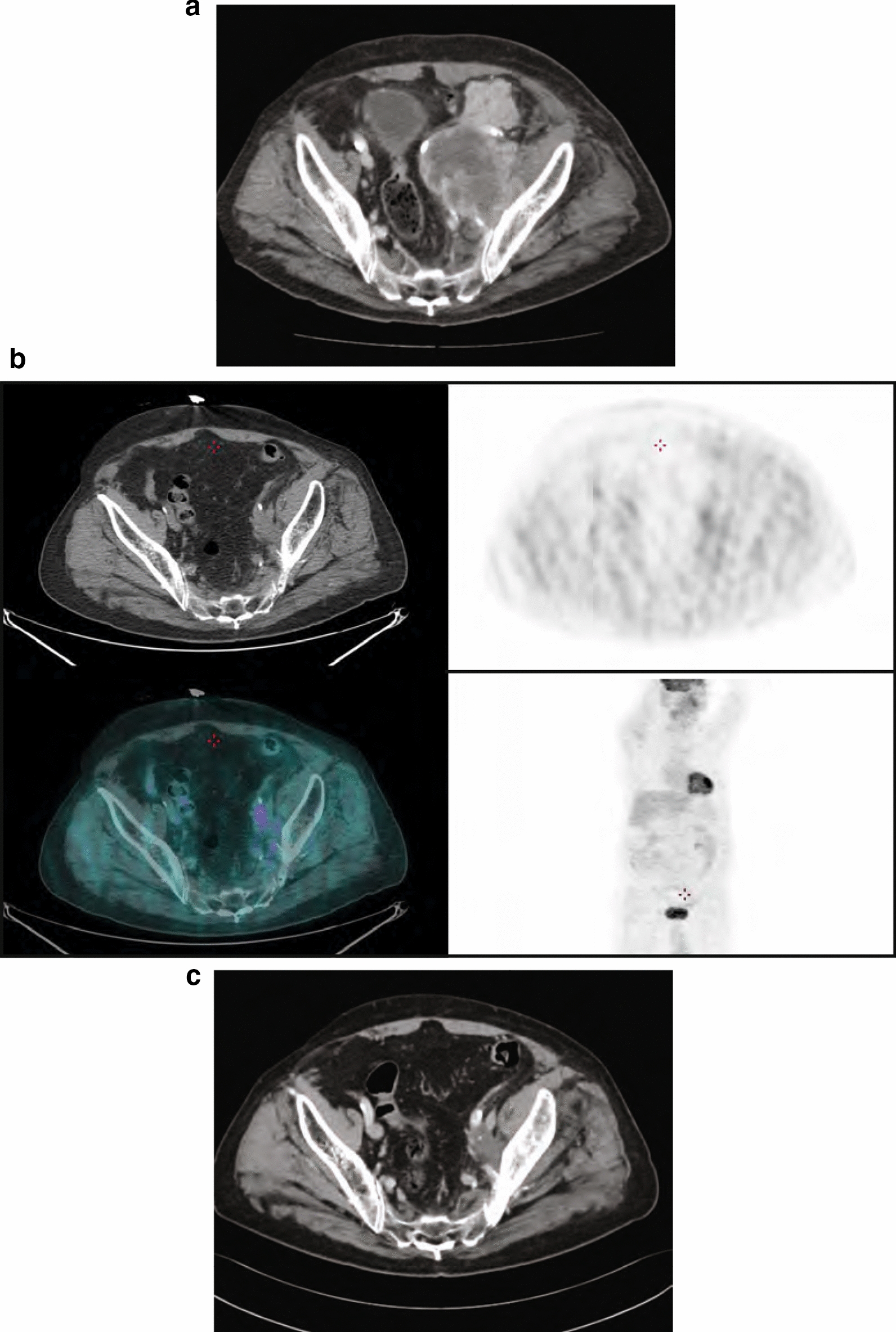
CT/PET-CT scans from a patient with metastatic Merkel cell carcinoma treated with avelumab. **a** Progressive disease at baseline (January 2017), **b** CR at 20 months after starting avelumab treatment (September 2018), and **c** CR in June 2020. Images were provided by Dr. Ciliberto. *CR* complete response, *PET-CT* positron emission tomography–computed tomography

**Fig. 6:**
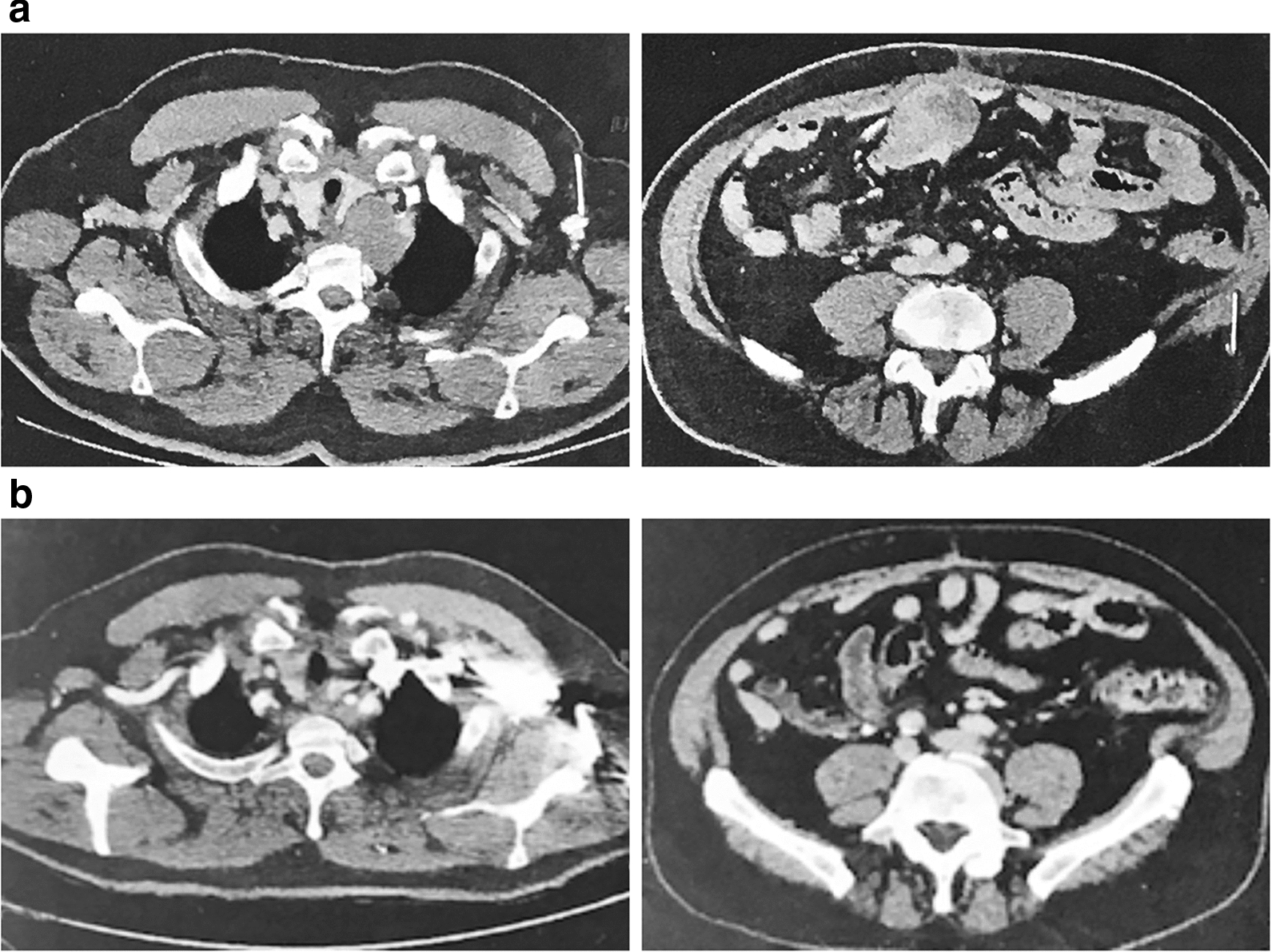
Computed tomography scans from a patient with metastatic Merkel cell carcinoma treated with avelumab. **a** Baseline and **b** complete response at 2 months after starting avelumab treatment. Images were provided by Dr. Corsi

Of 95 patients who received a supply of avelumab, response data were available for 55 patients (57.9%). In these 55 response-evaluable patients, the ORR was 29.1%, including CR in 6 (10.9%) and partial response in 10 (18.2%); 17 patients (30.9%) had stable disease as best overall response (Table 2). As a proportion of the total population supplied with avelumab (n = 95), the proportion who had an objective response was 16.8%. Images of tumor changes in avelumab-treated patients are shown in Figs. 1, 2, 3, 4, 5 and 6. Duration of avelumab treatment (or duration that drug was supplied) was assessed as an alternative measure of duration of clinical benefit because resupply required documentation of clinical benefit by the treating physician. At data cutoff, the median treatment duration was 9.7 months (range, 3.5–41.7 months).

**Table 2:** Physician-reported responses in all evaluable patients enrolled in Italy in the avelumab MCC EAP

Response parameter^a^	n = 55
ORR, %	29.1
DCR, %^b^	60.0
Confirmed BOR, n (%)	
CR	6 (10.9)
PR	10 (18.2)
SD	17 (30.9)
PD^c^	22 (40.0)
Duration of treatment for patients with response^d^	
Median (range), months	9.7 (3.5–41.7)

*BOR* best overall response, *CR* complete response, *DCR* disease control rate, *EAP* expanded access program, *MCC* Merkel cell carcinoma, *PD* progressive disease, *PR* partial response, *SD* stable disease

^a^Response was reported according to treating-physician assessment of follow-up scans at the time of resupply

^b^Among patients treated for a minimum of 3 months with available data

^c^Patients with PD or adverse events that required treatment discontinuation within the first 90 days were never resupplied and did not have a follow-up response evaluation; thus, these values may be underreported

^d^Duration of avelumab treatment/drug supply is reported as a surrogate for duration of response or clinical benefit

Physician-reported safety data are summarized in Table 3. The most frequently reported treatment-related AEs (TRAEs) were infusion-related reaction (single preferred term, n = 3 [3.2%]) and pyrexia (n = 2 [2.1%]). One immune-related AE was reported (myasthenia gravis [1.1%]). Safety data were reported at the treating physician’s discretion at the time of resupply, and many patients had no evaluable data beyond the 3-month supply; therefore, safety events were likely underreported.

**Table 3:** Physician-reported TRAEs in patients treated with avelumab in Italy in the MCC EAP

TRAEs, n (%)^a^	Treated patients (n = 95)		
	Nonserious events	Serious events	Total events
Infusion-related TRAEs^b^			
Infusion-related reaction^c^	1 (1.1)	2 (2.1)	3 (3.2)
Pyrexia	1 (1.1)	1 (1.1)	2 (2.1)
Anaphylactic reaction	0	1 (1.1)	1 (1.1)
Immune-related TRAEs^d^			
Myasthenia gravis	1 (1.1)	0	1 (1.1)
Other TRAEs			
Anemia	1 (1.1)	0	1 (1.1)
Urinary tract infection	1 (1.1)	0	1 (1.1)
Hyperglycemia	1 (1.1)	0	1 (1.1)

*AE* adverse event, *EAP* expanded access program, *MCC* Merkel cell carcinoma, *TRAE* treatment-related adverse event

^a^Data shown are preferred terms of all TRAEs observed in all patients enrolled from Italy in the EAP extracted from the safety database, including unsolicited cumulative events provided by treating physicians; overall safety events may have been underreported in this ad hoc program

^b^Infusion-related AEs based on a prespecified list of Medical Dictionary for Regulatory Activities preferred terms

^c^Infusion-related reaction based on the single Medical Dictionary for Regulatory Activities preferred term

^d^Immune-related AE based on medical review

Patients who achieved a CR with avelumab (n = 6) were investigated in detail, including additional follow-up beyond the cutoff date for the whole cohort (last follow-up in patients with CR, April to July 2020) (Table 4). Patients were aged 68 to 80 years, all were male, Eastern Cooperative Oncology Group performance status was 0 or 1, and 2 patients had diabetes mellitus as a comorbidity. All patients had PD after prior chemotherapy for mMCC. Time from start of treatment to CR ranged from 1.5 to 22 months, including confirmation of metabolic CR in 2 patients. After achieving a CR with avelumab, no patient required additional local or systemic anticancer therapy, and no patient developed new lesions. TRAEs among the 6 patients were grade 1/2 only (n = 4), resolved with acetaminophen (n = 1), or did not require treatment (n = 2).

**Table 4:** Summary of Italian patients who achieved a complete response in the avelumab MCC EAP

	Patient 1	Patient 2	Patient 3	Patient 4	Patient 5	Patient 6
Previous treatment prior to EAP enrollment	Surgery; radiotherapy; chemotherapy (cisplatin)	Surgery; radiotherapy; chemotherapy (carboplatin and etoposide)	Chemotherapy (cisplatin and etoposide)	Radiotherapy; chemotherapy (cisplatin-containing)	Surgery; radiotherapy; chemotherapy (cisplatin and etoposide)	Chemotherapy (carboplatin and etoposide)
Age at start of avelumab, years	79	76	71	80	70	66
Sex	Male	Male	Male	Male	Male	Male
ECOG PS	1	0	0	1	0	0
Major comorbidities or immunosuppressive conditions	None	Type 2 diabetes mellitus	None	Type 2 diabetes mellitus; hypertension; benign prostatic hyperplasia; glaucoma	None	None
Metastatic sites	Hepatic hilum; common iliac; right thigh and leg lymphedema	Retroperitoneal lymph nodes	Mediastinal and paratracheal lymph nodes	Lymph nodes and retroperitoneum	Thoracic, supraclavicular and inguinal lymph nodes; mesenteric tissue; right paraumbilical area	Abdomen (pancreas, stomach, spleen, and colon)
Date of first avelumab dose	August 22, 2017	May 2017	October 25, 2017	February 20, 2017	December 14, 2016	May 17, 2018
Date of documented CR	October 2, 2017 (including metabolic CR)	March 2019	March 14, 2018 (including metabolic CR)	December 9, 2017	February 2, 2017	August 28, 2018
Treatment-related toxicity	Transient subclinical hypothyroidism (not requiring treatment)	Pruritus (not requiring treatment); grade 1 asymptomatic pneumonitis (resolved with drug interruption)	Grade 2 pruritus	Grade 1 pruritus; potential correlation with basaloid penile intraepithelial neoplasia (surgically treated)	Slight fever and joint pain (resolved with acetaminophen)	Grade 1 thrombocytopenia (resolved without treatment)
New lesions with avelumab treatment	None	None	None	None	None	None
Date of last avelumab dose	September 6, 2019	February 2020	January 30, 2020	July 29, 2020	July 23, 2020	July 2, 2020
Additional local/systemic anticancer therapy	None	None	None	None	None	None
Date of last follow-up	April 27, 2020	July 13, 2020	May 12, 2020	July 29, 2020	October 12, 2020	July 2, 2020
Progression free at last follow-up	Yes	Yes	Yes	Yes	Yes	Yes

*CR* complete response, *EAP* expanded access program, *ECOG PS* Eastern Cooperative Oncology Group performance status, *MCC* Merkel cell carcinoma

## Discussion

The avelumab MCC EAP is the largest and only EAP ever opened for patients with this rare disease, enabling access to avelumab for patients with limited treatment options. The population of Italian patients reported included some patients who would not have been eligible for the pivotal JAVELIN Merkel 200 trial, including approximately 5% with an Eastern Cooperative Oncology Group performance status of 2 or 3. In addition, as reported previously for the overall global population [15], patients who were potentially immunocompromised were also eligible based on case-by-case assessment, although baseline comorbidities were not analyzed in detail for the Italian population. Data for PD-L1 expression and MCPyV status were not collected. All but 1 patient had received ≥ 1 prior line of chemotherapy before starting avelumab. The ORR in response-evaluable patients was similar to that reported in part A of the JAVELIN Merkel 200 trial, which enrolled only patients who had received ≥ 1 prior line of chemotherapy (29.1% vs. 33.0%, respectively) [11]. However, 40 patients were not evaluable for response because data were not available; unlike a clinical trial, data were submitted at the treating physician’s discretion and physicians often did not submit response data (administration of supplied avelumab to patients was also not confirmed). The ORR calculated using the denominator of the total population of Italian patients supplied with avelumab was 16.8%; therefore, the “true” ORR in this population may lie within the range of 16.8% to 29.1%. The most frequently reported TRAEs were infusion-related reaction and pyrexia, and no new safety signals were identified compared with previous studies.

Data collected in this EAP have various limitations compared with data obtained in a clinical trial. Safety and efficacy data for the EAP were reported at the treating physician’s discretion and are therefore potentially underreported. In this EAP population, median duration of treatment, which was assessed as an alternative measure of duration of clinical benefit, was 9.7 months, although it also likely represents an underestimate due to the nature of this EAP. In part A of JAVELIN Merkel 200, median progression-free survival was 2.7 months and median duration of response was 40.5 months [10, 11].

Positron emission tomography (PET), which captures metabolic changes in tumors by measuring fluorodeoxyglucose uptake, is increasingly being used to evaluate response to cancer treatment [16]. Metabolic changes due to malignancy or inflammation are generally detected earlier than the tumor structural changes that are captured by radiological imaging techniques, such as computed tomography [16, 17]. Studies in different tumor types have shown that a reduction in fluorodeoxyglucose uptake is associated with subsequent clinical and radiological responses to immunotherapy [17, 18]. Furthermore, complete metabolic tumor responses documented by PET have been shown to predict early response with immune checkpoint inhibitor treatment [19, 20] and may predict long-term benefit. In this EAP, 2 of the 6 patients with CR had confirmed metabolic CR by PET scan (documented in October 2017 and March 2018) and remained progression free at last follow-up (April 2020 and May 2020, respectively).

## Conclusions

The efficacy and safety of avelumab seen in an Italian real-world setting, including some patients who were ineligible for chemotherapy or clinical trial participation, support the findings of the JAVELIN Merkel 200 clinical trial and confirm avelumab as an active treatment option in patients with mMCC.

## Data Availability

For all new products or new indications approved in both the European Union and the United States after January 1, 2014, Merck KGaA, Darmstadt, Germany, will share patient-level and study-level data after deidentification, as well as redacted study protocols and clinical study reports from clinical trials in patients. These data will be shared with qualified scientific and medical researchers, upon researcher’s request, as necessary for conducting legitimate research. Such requests must be submitted in writing to the company’s data sharing portal. More information can be found at https://www.merckgroup.com/en/research/our-approach-to-research-and-development/healthcare/clinical-trials/commitment-responsible-data-sharing.html. Where Merck KGaA has a co-research, co-development, or co-marketing/co-promotion agreement or where the product has been outlicensed, it is recognized that the responsibility for disclosure may be dependent on the agreement between parties. Under these circumstances, Merck KGaA will endeavor to gain agreement to share data in response to requests.

## Abbreviations

AE: Adverse event
BOR: Best overall response
CR: Complete response
DCR: Disease control rate
EAP: Expanded access program
MCC: Merkel cell carcinoma
mMCC: Metastatic Merkel cell carcinoma
ORR: Objective response rate
PD: Progressive disease
PD-L1: Programmed death ligand 1
PET: Positron emission tomography
PR: Partial response
RECIST 1.1: Response Evaluation Criteria in Solid Tumors version 1.1
SD: Stable disease
TRAE: Treatment-related adverse event
